# miRNA-186 Improves Sepsis Induced Renal Injury Via PTEN/PI3K/AKT/P53 Pathway

**DOI:** 10.1515/med-2020-0036

**Published:** 2020-04-04

**Authors:** Min Li, Wei Li, Feng-Qin Ren, Ming-li Zhang

**Affiliations:** 1 Department of Intensive Care Unit, Jinan Central Hospital Affiliated to Shandong University, Jinan, Shandong, 250013, China

**Keywords:** miRNA-186, renal injury, apoptosis

## Abstract

**Aim:**

The aim of this study is to explain the effects of miRNA-186 in renal injury induced by sepsis.

**Methods:**

The Wistar rats were divided into 3 groups: the Sham group, Sepsis model group and the miRNA-186 group based on the model group; there were 9 rats in every group. The rat sepsis model was reproduced by cecal ligation and puncture (CLP). The rats of the miRNA-186 group were injected miRNA-186 from caudal vein. The rats of the difference group were killed after operation 24 h. The kidneys of the difference groups were taken for histopathological and cell apoptosis analysis by H&E and TUNEL assay. The relative protein expressions were measured by WB assay. miRNA-186 target to Phosphatase and tensin homologous protein (PTEN).

**Results:**

Compared with the Sham group, the kidney histopathological and cell apoptosis rates of the model group were significantly damaged (P<0.05, respectively), however, the kidney histopathological and cell apoptosis rate of miRNA-186 group were significantly improved compared with the model group (P<0.05, respectively). The relative protein expressions were significantly different among 3 groups (P<0.05, respectively). The PTEN was the target of the miRNA-186.

**Conclusion:**

miRNA-186 over-expression has effects that improve renal injury induced by sepsis via PTEN pathway.

## 1 Introduction

Acute kidney injury (AKI) is a clinical syndrome caused by a variety of causes of a sudden decline in renal function within a short period (hours or days). Severe infection and septic shock are among the most common causes of AKI, accounting for about 50% of acute renal failures (ARF) [1]. After AKI occurs, it will promote and aggravate other organs damage, leading to MODS (multiple organ dysfunction syndrome) and increased mortality. The fatality rate of severe infection complicated by ARF is 70%, which is obviously higher than that of ARF caused by other causes [2]. Recent studies have found that miRNA is involved in AKI apoptosis, inflammation, ischemia reperfusion (I/R) expression and regulation of angiogenesis and fibrosis, damage repair, and is closely related to the occurrence and development of AKI, the prognosis is considered to be the biological markers of early AKI [3, 4, 5]. The currently known AKI related miRNA including miR-21, miR-24, miR-122, miR-126, miR-210, miR-34a, miR-494, miR-92a, and miR-205 are involved in the occurrence, development and prognosis of AKI caused by various etiologies, and are of great significance for early intervention and treatment of patients [6, 7, 8, 9, 10, 11, 12, 13, 14]. In our present study, we firstly found that the miRNA-186 has an important role of PTEN targeting by biological software (http://www.targetscan.org/mamm_31/), we wanted to explain the effects of miRNA-186 in kidney injury induced by sepsis.

## 1 Material and methods

### 1.1 Animal and grouping

The Wistar male rat was 8 weeks of age, and the body weight was 220~250 g. The rats were purchased from Nanjing Medicine University. The surrounding environment is ventilated, the temperature is 22~25 degrees, and the humidity is from 50% to 60%. The rats were divided into 3 groups: the Sham group, the Model group and the miRNA-186 group. The sham group and the model group were injected with 0.5mL saline in the tail vein before and after operation. The rats of the miRNA-186 group were injected with miRNA-186. Before the experiment, they fasted overnight, drinking water freely. The rats were given 0.3% sodium pentobarbital (30mg/kg) intraperitoneal injection of anesthesia, routine abdominal iodophor disinfection, skin preparation and a sterile gauze hole towel. The skin of the abdominal wall was cut along the middle abdominal line at a length of about 1cm. The cecum was ligated with sterile forceps (ileum and cecum were kept away from the blood vessels in the middle of the cecum), the cecum was ligated with sterile targets and the head and tail of the cecum were perforated. The contents of the cecum were extruded out, the appendix and the contents were extruded back into the abdominal cavity together, and the abdominal wall incision was sutured. The animals in the sham operation group performed the same procedure without cecal ligation and perforation. The rats were killed 24h after the operation. The kidneys were dissected, the capsule was removed, weighed, and half of the kidney tissues were taken for microscopic examination. The remaining kidney tissue was placed in 10% formalin fixed, the urine and blood were collected.

### 1.2 Serum creatinine (Scr) and blood urea nitrogen (Bun) detection

Blood sample placed at room temperature for 1 h, Isolated serum as 3000r/min, stored at -20°C until measuring. The automatic multi-functional biochemical analyzer is used to determine the content in strict accordance with the requirements of the kit.

### 1.3 Pathological observation of kidney

The kidney tissues were fixed with 10% formalin, embedded and sliced with paraffin, and stained with HE staining. The pathological changes of kidney tissue were observed under a light microscope. 3 specimens were selected from each group, and 8 visual fields were randomly selected under optical microscope. 0: no damage; 1: renal tubular epithelial cells appeared inflammatory cell infiltration; 2 renal tubular large inflammatory cell infiltrations, lumen expansion; 3 renal tubular epithelial nuclei disappeared, dilated lumen; 4: destruction of renal tubular epithelial cells without nuclear staining structure.

### 1.4 TUNEL (TdT-mediated dUTP nick end labeling) assay

Paraffin sections of 5 μm thickness were stained with TUNEL. The nucleus changes and apoptosis of renal tissue were observed under a light microscope. The cells were stained brown as positive expression, and the apoptosis rate of renal tubular cells was calculated.

#### 1.4.1 WB (West Blotting) assay

The homogenizer fully grinded and homogenized the renal medulla tissue, lysis of RIRA cell lysate, placing 30min on the ice, centrifugation for 15min at 4°C by 14 000r/min, leaving the precipitate, and determining the protein concentration by BCA. A total protein of 30 g was obtained and electrophoresis was performed on 12% polyacrylamide (SDS-PAGE) gels, voltage 150V, 70min, at 4°C, 250mA current 1.5h transferred to PVDF film, 5% skimmed milk powder closed for 1h, respectively adding the goat anti rat PTEN, PI3K, AKT and P53 anti-body, cultured at 4°C overnight, removing the primary antibody, washing by TBST buffer solution at 3 times, 10 min/time, adding Corresponding horseradish peroxidase labeled second antibody, incubated at room temperature for 1.5h, ECL reagent color and exposure imaging, scanning images. The gray value of each band was analyzed by QuantityOne V6.42 software, and the ratio of the gray value of each target band with the ratio of gray value of glyceraldehyde-3-phosphate dehydrogenase (GAPDH) band in the same specimen was used as the semi quantitative result of the target protein.

### 1.5 Double luciferase reporter system analysis

The construction of wild type PTEN (PTEN 3’UTR-WT) and mutant (PTEN 3’UTR-MU) reporter gene vector, the reporter gene vector and miRNA-186 mimics and miRNA-186 inhibitor were co transfected into HK2 cells in each group which were co transfected to Renilla luciferase (PRL-TK) as reference, each experiment was replicated 3 times and 48 h after transfection, with each hole dual luciferase reagent in microplate chemiluminescence detection of firefly luciferase and Renilla luciferase activity analyzer.

### 1.6 Statistical analysis

All experimental data were analyzed by SPSS13.0 and expressed as mean ± standard deviation (mean±SD). Differences among multiple groups were compared by one-way analysis of variance.

**Ethical approval**: The research related to animals use has been complied with all the relevant national regulations and institutional policies forthe care and use of animals.

## 2 Results

### 2.1 Comparison of serum Cr and BUN levels in each group

The content of Scr and BUN in the sepsis group was significantly higher than that of the Sham group (P<0.05, respectively, Table 1). However, after the miRNA-186 injection, the Scr and BUN concentrations of the miRNA-186 group were significantly depressed compared with those of the Model group (P<0.05, respectively, Table 1).

**Table 1:** Comparison of serum creatinine and urea nitrogen levels in each group (Mean±SD)

Group	n	Scr (μmol/L)	BUN (mmol/L)
Sham	10	34.04±2.41	8.53±0.56
Model	10	54.77±2.70^*^	27.43±2.00^*^
miRNA-186	10	38.72±2.37^*^^#^	14.91±1.91^*^^#^

* P<0.05, compared with Sham group;

# P<0.05, compared with Model group

### 2.2 Pathological observation of kidney

**Figure 1:**
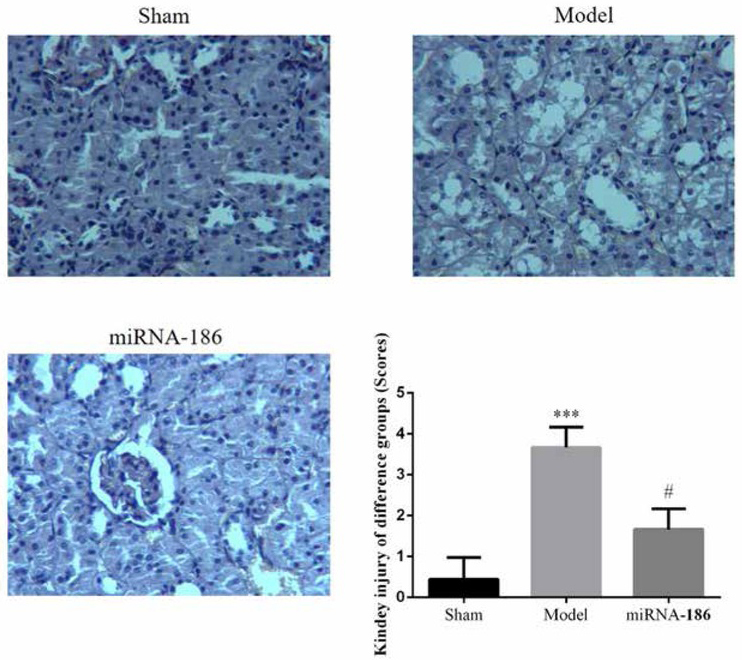
Pathological observation of difference groups, ***: P0.05, compared with Sham group, #: P<0.05, compared with Model group

Under the microscope for the sham operation group, the renal tissue structure is clear, renal tubules, renal interstitial edema and there is no sign of inflammatory cell infiltration; the renal tissue of the rats in the model group showed significant inflammatory pathological changes, increased glomerular volume, interstitial inflammatory cell infiltration and scattered bleeding points can be seen, renal tubular epithelial cells, vascular stenosis swelling and degeneration; The pathological changes of miRNA-186 group were improved, and the edema of epithelial cells and inflammatory cells in the stroma were significantly less than those in the model group. The kidney injury of miRNA-186 group was significantly reduced compared with model group (P<005). The data was shown in Figure 1.

### 2.3 Cell apoptosis of difference groups by TUNEL

**Figure 2:**
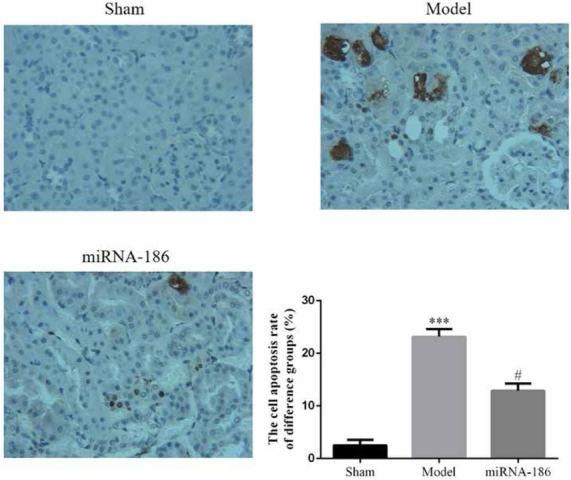
The cell apoptosis rate of difference groups, ***: P<0.05, compared with Sham group, #: P<0.05, compared with Model group

To investigate kidney cell apoptosis and the effects of miRNA-186 to improve cell apoptosis, we measured the cell apoptosis rate of difference groups by TUNEL assay. The results showed that the cell apoptosis of the model group was significantly enhanced compared with the sham group (P<0.05). However, the cell apoptosis rate of miRNA-186 group was significantly improved compared with the model group (P<0.05). The data were shown in Figure 2.

### 2.4 The relative proteins expression by WB assay

**Figure 3:**
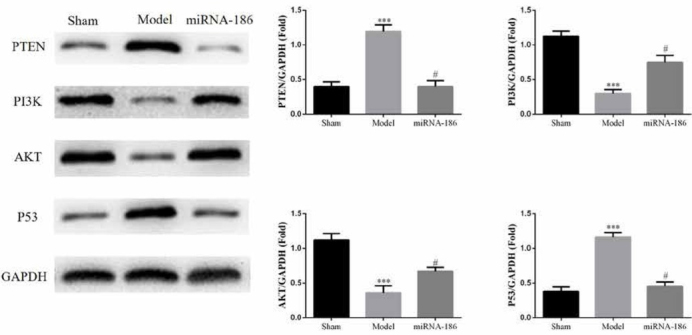
The relative proteins expressions of difference groups, ***: P<0.05, compared with Sham group, #: P<0.05, compared with Model group

Compared with the Sham group, the PTEN and P53 proteins expressions were significantly up-regulated (P<0.05, respectively) and the PI3K and AKT proteins expressions were significantly down-regulated (P<0.05, respectively) in the model group. However, the PTEN and P53 proteins expressions were significantly suppressed (P<0.05, respectively) and the PI3K and AKT proteins expressions were significantly enhanced (P<0.05, respectively) in the miRNA-186 group compared with those in the model group. The data were shown in Figure 3.

### 2.5 Double luciferase gene reporter

**Figure 4:**
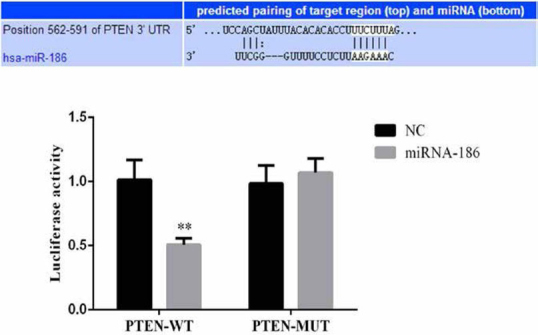
Dual luciferase target assay, **:P<0.05, compared with NC group

Double luciferase gene reporter experiments have shown that miRNA-186 can bind to the 3 ‘UTR region of PTEN, and the above experiments show that miRNA-186 can inhibit its expression by binding to PTEN’s 3’ UTR. The data were shown in Figure 4.

## 3 Discussion

Systemic infection causes acute kidney injury (AKI), accounting for about half of all AKI [15]. The fatality rate of severe infection complicated with acute renal failure is as high as 70% [16, 17]. At present the early treatment for septic shock worldwide continues to develop, such as the application and bundle treatment of early goal-directed therapy, intensive glycemic control, stress hormone therapy, activated protein C, but there is no clinical method that can effectively prevent AKI and shorten the course of the measures [18].

In recent years, the function of miRNAs research has turned more and more attention to people, mainly through animal source miRNAs and the target gene of mRNA 3 ‘non encoding region (3 UTR) is not fully or completely matched with each other, at the level of translation on the specific inhibition of gene expression. At present, the study of miRNAs are concentrated in the field of oncology [19, 20], meanwhile, some studies had shown that miRNAs also have effects that regulate kidney injury [21, 22]. The role of PTEN in inhibiting proliferation and promoting apoptosis has been demonstrated in a variety of tumor cells [23, 24, 25], and PTEN can effectively inhibit the expression of PI3K/AKT signaling pathway. PTEN, through its phosphatase activity, removesPIP3 third phosphate groups into PIP2, thereby inhibiting the PI3K/Akt signaling pathway, inhibit cell proliferation and promoting cell apoptosis and other biological effects [26, 27, 28]. The PI3K/AKT signaling pathway can negatively regulate P53 gene. P53 is an important gene downstream of PI3K/ AKT. P53 over-expression can induce cell cycle arrest and promote apoptosis [29, 30]. In our present study, we found that miRNA-186 over-expression had effects that improve kidney injury induced by sepsis. With miRNA-186 enhancing, the PTEN protein expression was suppressed, PI3K and AKT proteins expressions were stimulated and the P53 protein expression was down-regulated. Depending on those results, we inferred that miRNA-186 might be targeting PTEN. By dual luciferase target assay, the results were proved that miRNA-186 target PTEN.

In conclusion, miRNA-186 has effects that improve kidney injury induced by sepsis by regulation of PTEN/ PI3K/AKT/P53 signaling pathways in vivo study.
